# Assessing the Satisfaction and Acceptability of an Online Parent Coaching Intervention: A Mixed-Methods Approach

**DOI:** 10.3389/fpsyg.2022.859145

**Published:** 2022-07-28

**Authors:** Lu Qu, Huiying Chen, Haylie Miller, Alison Miller, Costanza Colombi, Weiyun Chen, Dale A. Ulrich

**Affiliations:** ^1^Institute of AI for Education, East China Normal University, Shanghai, China; ^2^School of Kinesiology, University of Michigan, Ann Arbor, MI, United States; ^3^College of Physical Education, Yangzhou University, Yangzhou, China; ^4^School of Public Health, University of Michigan, Ann Arbor, MI, United States; ^5^IRCCS Fondazione Stella Maris, Pisa, Italy

**Keywords:** parent training, autism, telehealth, parent coaching/mediated, home-based program, acceptability, satisfaction, mixed methods

## Abstract

**Background:**

Parent-mediated intervention (PMI) has been studied in promoting skill acquisition or behavior change in the children with autism spectrum disorder. Most studies emphasize on the improvement of child’s core symptoms or maladaptive behaviors, making parental perceived competence and self-efficacy secondary. Yet, the evaluations of intervention implementation are under-reported, especially when translating such interventions into a new population or context. This research investigated the intervention implementation of a 12-week parent coaching intervention which was delivered through telehealth and tailored to Chinese population. The intervention was based on the Parent-mediated Early Start Denver Model with culturally adapted lectures, manuals, and demonstration and commentary videos. This study aimed to evaluate the intervention implementation by assessing parents’ satisfaction, acceptability, appropriateness, and feasibility.

**Method:**

A randomized controlled trial was conducted with two telehealth conditions: self-directed and web+group therapy. Parents in the self-directed condition received intervention individually through the online learning platform. The web+group therapy condition navigated the same program with weekly 1.5-h group coaching sessions *via* videoconferencing. This mixed-methods study used a concurrent convergent design to evaluate the intervention implementation at post-intervention. The quantitative data was collected from the Program Evaluation Survey and the qualitative data was collected from five focus groups.

**Results:**

Parents in self-directed group reported significantly lower scores in total perceived competence than parents in web+group therapy condition, while there was no group difference on the total self-efficacy. *Tailored feedback, demonstration and commentary videos, peer commenting, live coaching*, and *guided reflection* were the top-five acceptable telehealth strategies that were strongly endorsed by parents. *Family centered care, home-based intervention, strategies relative to daily activities, the remote learning platform*, and *the program-based community* were elements that parents considered when evaluating the program’s appropriateness. *Parent modeling, step-by-step instructions, and tailored feedback* were key components in making intervention strategies feasible for parents to implement at home.

**Conclusion:**

Findings indicate the application of telehealth was acceptable, appropriate, and feasible for Chinese parents. Group-based parent coaching intervention *via* videoconferencing could be a promising home-based service model to increase parental perceived competence. A large-scale RCT is needed to investigate the effectiveness of group-based PMI *via* telehealth.

## Introduction

Early intensive behavioral intervention (EIBI) is a common treatment based on applied behavior analysis principles for young children with autism spectrum disorder (ASD; Center, 2009, 2015; Eldevik et al., 2009; Weisz and Kazdin, 2010). Despite the potential long-term contribution to lowering special education (Chasson et al., 2007) or lifetime care costs (Piccininni et al., 2017), EIBI has a high demand for trained specialists, and the cost of EIBI services for each family remains high (Jacobson et al., 1998). Systematic barriers, such as a shortage of trained professionals (Liao et al., 2021), disjointed care coordination (Vohra et al., 2014), and high service cost (Vohra et al., 2014; Sobotka et al., 2016), result in insufficient access to EIBI. To help overcome these barriers, the involvement of parents in implementing intervention strategies allows intervention to begin early and sustain (Green et al., 2015, 2017). Research suggests that parents’ active involvement in the diagnostic and therapeutic processes could benefit their children with developmental disorders, particularly in young children with ASD. Parent training and education provided intervention strategies and allowed parents to remain highly engaged in their children’s education and development (Chaidi and Drigas, 2020).

Parent-mediated intervention (PMI) is an effective evidence-based practice (EBP) which was identified by the National Professional Development Center on Autism Spectrum Disorder (NPDC; Wong et al., 2015). PMI focuses on teaching parents how to promote social interaction, imitation, communication, and play skills for their children with ASD in natural environments (Oono et al., 2013; Bearss et al., 2015). Systematic reviews suggested the benefit of PMIs in children with ASD included motivation to interact, joint attention, imitation, non-verbal, and verbal communication (Oono et al., 2013; Estes et al., 2014; Nevill et al., 2018; Jurek et al., 2021). PMIs have been widely adopted for very young children with ASD, such as JASPER (Kasari et al., 2006, 2010), Project ImPACT (Ingersoll and Wainer, 2013; Stadnick et al., 2015), Parent-mediated Early Start Denver Model (P-ESDM; Rogers et al., 2012; Vismara et al., 2013). PMIs have shown promising effectiveness (Deb et al., 2020) in home settings (Siller et al., 2013; Poslawsky et al., 2015), clinical settings (Green et al., 2010), community settings (Eapen et al., 2013; Stadnick et al., 2015; Edwards et al., 2019), and school settings (Goods et al., 2013). Liu et al. (2020) systematically reviewed 21 PMIs in China and concluded that the identified programs lacked the capacity to be further disseminated in Chinese societies due to a lack of underlying theoretical frameworks, under-reported implementation outcomes, and the insufficiency of cultural adaptations.

Families of a child with ASD in China face two key challenges: limited access to appropriate health services and a shortage of trained professionals. In China, 0.70–1.00% of the population is diagnosed with autism (Sun et al., 2013b,^2019^; Zhou et al., 2020). According to the Seventh National Population Census in China (Ning, 2021), there were 253.38 million children under 14 years of age, this translates to about 1.75–2.53 million children with ASD who need access to pediatricians and rehabilitation-related therapists. However, the number of pediatricians and rehabilitation-related therapists in China was only about 100,000 and 30,000, respectively, in 2015 (Wu et al., 2015). The shortage of trained specialists in mainland China (McCabe, 2012; Hu and Yang, 2013) hampered the delivery of early intervention services, resulting in a long waitlist for enrollment and high-priced programs (Chang and Zaroff, 2017; Zhou et al., 2018). The scarcity of early intervention services in China reflects a massive imbalance between the number of professionals qualified to provide EBP treatments and patients in need. Such conditions call for research on innovative service models and delivery formats to address issues mentioned above.

The Early Start Denver Model (ESDM) is an EBP designed for young children with ASD aged 1 to 5. It adopted behavioral and developmental principles to promote overall development and autism-related outcomes (Rogers and Dawson, 2010). The P-ESDM, developed by Rogers et al. (2012), focused on the promotion of social interaction and communication between children with ASD and their caregivers in a natural environment. The natural environments enable parents to learn skills or strategies at their familiar surroundings and to use available toys and resources to stimulate the child develop and grow (Catalino and Meyer, 2016). Such a family centered service in natural environments facilitates caregivers to learn how to integrate intervention strategies in their children’s everyday routines and activities (Trivette and Keilty, 2017). P-ESDM has the potential to be delivered successfully through telehealth methods. Vismara et al. (2012) examined the telehealth delivery of P-ESDM and suggested that parents were successful in learning similar content *via* telehealth. The remote coaching allowed parents to use strategies more often in their daily interactions with children. Two studies examined the effectiveness of P-ESDM on preschoolers with ASD in mainland China. Xu et al. (2017) examined the efficacy of an 8-week ESDM plus other eclectic intervention services delivered by local teachers. Zhou et al. (2018) conducted a non-randomized trial to compare a 26-week, high-intensity, P-ESDM intervention with a general community-based treatment program. Both studies were conducted in a clinical in-person setting and showed positive results in participating children. However, those two studies did not provide information on the program implementation or on parents’ competence. The evaluation of program implementation, such as acceptability, appropriateness, and feasibility, is critical when translating existing EBPs into a different cultural context (Karsh, 2004; Proctor et al., 2011). There is a gap in the literature to conduct pilot evaluations on the parental acceptability, cultural appropriateness, and treatment feasibility in the Chinese context.

Telehealth could be a potential service delivery solution to address limited access for families in China. Telehealth is defined as a provision of health care remotely through the usage of a variety of telecommunication tools, such as telephones, smartphones, and mobile wireless devices, with or without a video connection (Dorsey and Topol, 2016). The application of telehealth in providing education sessions has been widely adopted when delivering PMI to parents of young children with ASD. Parsons et al. (2017) systematically reviewed parent training programs which were delivered remotely for children with ASD living outside urban areas. Preliminary evidence suggested that remotely delivered PMI training may improve caregiver expertise, enhance intervention fidelity, and improve social and communication skills in children with ASD. Among those seven studies included in the systematic review, there were only two randomized controlled trials (Ingersoll and Berger, 2015; Ingersoll et al., 2016). Pickard et al. (2016) suggested that the telehealth could be an efficient delivery model for evidence-based parent training interventions.

Group-based parent training may help to offset the shortage of professionals in China who specialize in autism-related services. O’Donovan et al. (2019) conducted a systematic review to examine group-based parent training interventions for families of children with ASD. According to their findings, group-based parent training interventions improved parent behavior, children’s behavior, parent health, and social support. However, with 12 out of 13 included studies falling below the quality rating threshold, O’Donovan et al. (2019) stated that those findings should to be interpreted with caution and be further investigated using better study design and methodology. Banbury et al. (2018) found that health professional-led group videoconferencing was a feasible option for that to provide education and social support into the home setting. Group-based interventions *via* videoconferencing enhanced accessibility, and the effects of the videoconferencing groups were comparable to those of in-person groups (Banbury et al., 2018).

During the recent COVID-19 pandemic, there was a trend in the use of telehealth to deliver services for young children with ASD and their families, such as social communication interventions (Simacek et al., 2021), diagnostic assessments (Gibbs et al., 2021), early interventions (Pickard et al., 2016; Mullan et al., 2021), and ABA related services (Ferguson et al., 2019). Therefore, we developed a culturally adapted group-based parent coaching intervention based on the P-ESDM. We created a 12-week telehealth program to support families with a newly diagnosed child in mainland China. The program taught family caregivers how to integrate evidence-based intervention strategies into everyday routines and home activities. A randomized controlled feasibility trial was conducted with two telehealth conditions, a self-directed and web+group therapy conditions. The self-directed condition received intervention individually through the online learning platform. The web+group therapy condition (treatment) navigated the same program as the self-directed condition (comparison) with a weekly 1.5-h group coaching therapy *via* videoconferencing.

The primary goal of the present study was not determining if this intervention was effective by investigating the child or parent outcome measures, but rather, how the end-users of this culturally adapted telehealth intervention, parents of children with ASD, evaluated the intervention implementation. We adopted a mixed-methods approach in the program evaluation because it enabled researchers to engage different stakeholders, such as parents, to evaluate the intervention implementation (Nastasi et al., 2007). The mixed-methods research approaches were widely used in the health science, especially when translating EBPs into a different population or adopting culturally competent practices (Nastasi et al., 2007; Creswell et al., 2011). The reason for using a mixed methods approach is that it enables us to ask research questions that call from a real-world intervention study by adding multiple perspectives together to enhance and enrich the meaning of a singular perspective (Creswell et al., 2011). For example, the mixed methods design provides insight into qualitatively explaining the implementation outcomes with qualitative data, the merge of quantitative and qualitative data can develop a more complete understanding of constructs in the program evaluation. The findings of this mixed methods study could in turn facilitate the refinement and distribution of this culturally adapted telehealth intervention in the future.

The current study followed “the conceptual framework for implementation outcomes” (Proctor et al., 2011) to evaluate the implementation effectiveness. Acceptability, appropriateness, and feasibility were constructs which measured the implementation from the patients’ level. Moreover, acceptability, appropriateness, and feasibility were considered as “leading indicators” of implementation success (Weiner et al., 2017). The evaluation of intervention implementation could facilitate researchers to distinguish if the failure occurred in the implementation process or the intervention was ineffective in the new context. Therefore, it is essential to test the satisfaction and acceptability from parents’ perspectives about the program content and telehealth delivery when translating an EBP (P-ESDM) in a new context (the Chinese community). Furthermore, the perceptions of parents may facilitate a better understanding about the experience of these families and inform future development, adoption, and implementation of such intervention into a new population.

Overall, the present study used a concurrent convergent mixed-methods design to evaluate Chinese parents’ perceptions regarding their participation in a 12-week culturally adapted group-based parent coaching intervention *via* telehealth. The intervention implementation was evaluated quantitatively using a program evaluation survey and qualitatively with focus group interviews conducted immediately after the intervention. The intervention implementation was assessed in parents’ satisfaction, acceptability, appropriateness, and feasibility. The quantitative objective was to determine if the two telehealth conditions evaluated the program implementation differently. The qualitative objective was to obtain the nuance understanding about parents’ perceptions about their 12-week experience in this telehealth intervention. The mixed-methods objective was to merge the qualitative and quantitative findings and generate integrated findings to obtain a more comprehensive evaluation on the implementation of this culturally adapted telehealth intervention from Chinese parents’ perspectives.

## Materials and Methods

### Study Design and Ethical Approval

The current study used a concurrent convergent design and analyzed the post-intervention program evaluation data from a group-based parent coaching intervention delivered *via* telehealth. The study design diagram is presented in Table 1. To obtain a comprehensive understanding from the parent perspective, the mixed methods integration strategies included *matching* (Fetters et al., 2013) survey questions to focus group prompts and *merging* (Fetters et al., 2013) the qualitative and quantitative findings through a side-by-side joint display (Guetterman et al., 2015) with meta-inferences and an assessment of fit.

**TABLE 1:** Convergent mixed methods design diagram.

	Quantitative		Qualitative
Participant	*N* = 32		*N* = 24
Data resource	Program evaluation survey Close-ended questions 32 items	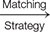	Five focus group interviews Open-ended questions 15 prompts
Analytic approach	Independent *t*-test		Thematic analysis
Data integration	Merging strategy: Statistics by theme joint display		

Ethical approval was granted in July 2020 by the University of Michigan Health Sciences and Behavioral Sciences Institutional Review Board (HUM00182526). The intervention study was conducted from September 7th, 2020, to January 17th, 2021. The post-intervention program evaluation data was collected from November 30th to December 12th, 2020.

### Participants

Thirty-two primary caregivers who completed the post-intervention Program Evaluation Survey from the intervention study were selected in this study. The inclusion criteria for participating the intervention study were: (1) parents over age of 18, (2) with a child aged 2–5 years old with a diagnosis of ASD made by a psychiatrist within 6 months at entry, (3) have access to the Internet and a digital device to receive the web-based intervention, and (4) the participating child met or exceeded cutoff scores of 10 (social interaction), 8 (communication and language), and 3 (restricted and repetitive patterns of behavior or interest) on the Autism Diagnostic Interview-Revised (Lord et al., 1994). Participants were recruited online through parent groups and community service providers.

There were 18 participants in the group-based treatment group (web+group therapy) and 14 participants in the active comparison group (self-directed). Parents were randomly assigned to the treatment or comparison group. All participants received informed consent prior to participation in the intervention study and focus group, 24 of 32 agreed to participate in the focus group. Not all participants completed all the modules at post-intervention assessment; the *intervention completion ratio* was calculated by dividing the number of participants who completed the 12-week intervention by the total number of participants in the group. Table 2 displays the participants’ demographic characteristics.

**TABLE 2:** Demographic characteristics of participants in program evaluation survey and focus group interview.

Characteristics	Program evaluation survey			Focus group interview		
	Self-directed (*n* = 14)	Web+group therapy (*n* = 18)	Total (*n* = 32)	Self-directed (*n* = 7)	Web+group therapy (*n* = 17)	Total (*n* = 24)
Participant relation, *N* (%)						
Mother	13 (92.86)	14 (77.78)	27 (84.38)	7 (100.00)	13 (76.47)	20 (83.33)
Adoptive mother	0 (0.00)	1 (5.56)	1 (3.13)	0 (0.00)	1 (5.88)	1 (4.17)
Father	1 (7.14)	3 (16.67)	4 (12.50)	0 (0.00)	3 (17.65)	3 (12.50)
Intervention completion ratio						
Sample ratio	8:14 (0.57)	17:18 (0.94)	25:32 (0.78)	6:7 (0.85)	16:17 (0.94)	22:24 (0.91)
Child Age (years),						
Mean ± SD	3.08 ± 0.77	3.23 ± 0.98	3.16 ± 0.88	3.34 ± 0.87	3.29 ± 0.97	3.31 ± 0.93
Range	1.68–4.62	1.70–5.16	1.68–5.16	2.37–4.62	1.70–5.16	1.70–5.16
Child Sex, *N* (%)						
Males	12 (85.71)	15 (83.33)	27 (84.38)	6 (85.71)	14 (82.35)	20 (83.33)
Females	2 (14.29)	3 (16.67)	5 (15.63)	1 (14.29)	3 (17.65)	4 (16.67)
Child verbal, N (%)						
Non-verbal	9 (64.29)	12 (66.67)	21 (65.63)	3 (42.86)	11 (64.71)	14 (58.33)
Verbal	5 (35.71)	6 (33.33)	11 (34.38)	4 (57.14)	6 (35.29)	10 (41.67)
ADI-R, Mean ± SD						
Social impairment	20.86 ± 3.39	19.39 ± 4.24	20.03 ± 3.91	20.86 ± 4.10	19.24 ± 4.32	19.71 ± 4.24
Communication	10.79 ± 1.97	9.44 ± 2.89	10.03 ± 2.58	10.71 ± 2.43	9.47 ± 2.98	9.83 ± 2.84
Repetitive interest	3.50 ± 1.83	3.44 ± 1.95	3.47 ± 1.87	3.86 ± 1.95	3.35 ± 1.97	3.50 ± 1.93
Total	9.29 ± 3.17	7.22 ± 3.93	8.13 ± 3.71	9.29 ± 2.56	7.12 ± 4.03	7.75 ± 3.74
Family structure, *N* (%)						
Nuclear family	5 (35.71)	3 (16.67)	8 (25.00)	2 (28.57)	3 (17.65)	5 (20.83)
Three generations family	9 (64.29)	14 (77.78)	23 (71.88)	5 (71.43)	13 (76.47)	18 (75.00)
Other	0 (0.00)	1 (5.56)	1 (3.13)	0 (0.00)	1 (5.88)	1 (4.17)
Monthly family income, *N* (%)						
<460 Dollars	1 (7.14)	1 (5.56)	2 (6.25)	0 (0.00)	1 (5.88)	1 (4.17)
460–770 Dollars	0 (0.00)	2 (11.11)	2 (6.25)	0 (0.00)	2 (11.76)	2 (8.33)
770–1,540 Dollars	4 (28.57)	4 (22.22)	8 (25.00)	2 (28.57)	4 (23.53)	6 (25.00)
1,540–3,080 Dollars	6 (42.86)	7 (38.89)	13 (40.63)	2 (28.57)	6 (35.29)	8 (33.33)
3,080–7,690 Dollars	3 (21.43)	4 (22.22)	7 (21.88)	3 (42.86)	4 (23.53)	7 (29.17)
Location, *N* (%)						
Urban	12 (85.71)	15 (83.33)	27 (84.38)	6 (85.71)	14 (82.35)	20 (83.33)
Rural	2 (14.29)	3 (16.67)	5 (15.63)	1 (14.29)	3 (17.65)	4 (16.67)
Mother’s education, *N* (%)						
High school or below	1 (7.14)	1 (5.56)	2 (6.25)	0 (0.00)	1 (5.88)	1 (4.17)
Junior college	4 (28.57)	6 (33.33)	10 (31.25)	3 (42.86)	6 (35.29)	9 (37.50)
Undergraduate	7 (50.00)	8 (44.44)	15 (46.88)	2 (28.57)	7 (41.18)	9 (37.50)
Graduate or above	2 (14.29)	3 (16.67)	5 (15.63)	2 (28.57)	3 (17.65)	5 (20.83)
Mother’s employment, *N* (%)						
Full-time	5 (35.71)	10 (55.56)	15 (46.88)	2 (28.57)	9 (52.94)	11 (45.83)
Part-time	0 (0.00)	1 (5.56)	1 (3.13)	0 (0.00)	1 (5.88)	1 (4.17)
Unemployed	9 (64.29)	6 (33.33)	15 (46.88)	5 (71.43)	6 (35.29)	11 (45.83)
Self-employed	0 (0.00)	1 (5.56)	1 (3.13)	0 (0.00)	1 (5.88)	1 (4.17)

### Intervention

The Group-based Parent Coaching Intervention is a culturally adapted program that uses the P-ESDM as the structure (Vismara et al., 2009, 2012, 2013). The 12-week program was delivered in Chinese *via* the Canvas online learning management system, and each module covered one of the following topics: (1) Autism and what to expect, (2) Increasing the child’s attention, (3) Sensory social routines, (4) Dyadic engagement, (5) Non-verbal communication, (6) Imitation, (7) ABC’s of learning, (8) Joint attention, (9) Play, (10) Pretend play, (11) Speech development, and (12) Program summary. To fit the knowledge level and demands of Chinese parents, the program was modified using a family capacity-building approach with four types of intervention materials. They were: (1) asynchronous lectures with a practice manual, (2) demonstration and commentary videos, (3) web-based Q&A session, and (4) homework (optional). The *Lectures* were asynchronous videos that emphasized the understanding and application of intervention strategies for young children with ASD. Each module included *a practice manual* with instructions and reflection questions to help parents practice on their own at home. The *Demonstration videos* were authentic video vignettes of parents interacting with their children while implementing intervention strategies, while the *Commentary videos* highlighted the timing and key characteristics of the practices. Activities in the *Demonstration videos* were culturally appropriate and had a Chinese subtitle. Parents were encouraged to discuss and share their experiences in a *Q&A session* on the Canvas website. Parents voluntarily submitted *homework* each week, and those who did received individual written feedback from the therapist.

During the intervention, the active comparison group (self-directed) navigated the program on their own pace, whereas the treatment group (web+group therapy) followed the same program but also participated an additional 1.5-hour virtual *group therapy session* every week. The *weekly group therapy* was a synchronous group-based parent coaching session *via* telehealth. Each session lasted 1.5 hours and followed a standardized protocol to guide reflection *via* a videoconferencing software, DingTalk. The program was designed under the supervision of a senior licensed ESDM trainer who is also a clinical psychiatrist and delivered by a licensed physical therapist who is a native Chinese and has completed ESDM Introduction and Advanced trainings.

### Measures

#### Demographics

Basic demographic information was reported by parents including age, sex, education level, employment status, family income and structure, as well as the age and gender of their children with ASD.

#### Quantitative Evaluation: Program Evaluation Survey

The post-intervention Program Evaluation Survey was developed to evaluate (1) parents’ perceptions of the program implementation, and (2) perceived competence and self-efficacy. The first 12 items (item1–12) focused on the program implementation, while the remaining 20 items (item 13–32) focused on parents’ perceived competence and self-efficacy in each week’s skills. The survey used a 5-point Likert scale, with responses ranging from 5 (very agree) to 1 (very disagree). Item 1–12 evaluated the program in the following aspects: satisfaction, recommendation, acceptability, appropriateness, feasibility, program difficulty, background knowledge, and frequency of use. Acceptability, appropriateness, and feasibility items were extracted and modified from the published measures with strong psychometric properties, the Acceptability of Intervention Measure, Intervention Appropriateness Measure, and Feasibility of Intervention Measure (Weiner et al., 2017). The items from these three Measures mentioned were translated to Chinese by two bilingual research assistants independently and translated back to English by another bilingual colleague. The translation and translation-back processes were conducted by persons who were not involved in this study. Satisfaction, acceptability, appropriateness, and feasibility had two items each, assessing both the program content and telehealth delivery. In addition to those four constructs, we asked participants to rate the difficulty level of the program, the need for background knowledge, the frequency of use, and their recommendation for improving future iterations of the program (see Table 3, for the survey constructs). Item 13–32 asked parents about their perceived competence and self-efficacy in relation to the skills of the week. *Total perceived competence* and *total self-efficacy* were calculated by adding the scores from each week, with a maximal of 50 (see Supplementary Appendix A for original survey questions). According to the results of the Cronbach’s alpha analysis, the Program Evaluation Survey indicated excellent internal reliability (α = 0.95). Specifically, the Cronbach’s alpha for the implementation questions (item 1–12) was good, α = 0.84, and excellent (α = 0.96) for questions on the perceived competence and self-efficacy (item 13–32). All items on the scale appeared to be worthy of retention, resulting in a decrease in alpha if deleted.

**TABLE 3:** Examples of matching survey questions to focus group prompts.

Program evaluation survey	Focus group prompts
Program implementation Satisfaction (1) Recommendation (15) Acceptability (2,3) Appropriateness (4,5,11) Feasibility (2,3,4,5,11,12) Program Difficulty (6,8) Background Needed (9) Frequency (7) Perceived competence and self-efficacy Perceived Competence (13) Self-efficacy (13)	1. What is your overall perception of this web-based program? For example, the program content, Telehealth format. 2. What are your favorite parts of this web-based program? For example, topics covered in the program, learning materials, how the program run, etc. 3. What do you dislike most about this web-based program? For example, topics covered in the program, learning materials, how the program run, etc. 4. What aspects of this web-based program do you think meet your expectations? Or what aspects meet the needs of you and your family? 5. What are your favorite and least favorite parts about the remote learning? And Why? e.g., compared with in person learning? 6. During the intervention, which parts do you find difficult to learn? Which parts are relatively easy? 7. What do you feel about the intensity of current program? Do you think you can keep up or not? Any reasons for that? 8. What barriers did you experience during the intervention? Any difficulties? Or burden? For example, time, energy, participation, software, and hardware, etc. 9. What did you think about having a coach? Is it necessary? In the beginning or later? 10. What if, the program is not funded by research, are you willing to participate? e.g., Any considerations? 11. (Treatment group) What did you think about the group session? What are your favorite and least favorite parts? 12. (Treatment group) What support do you get from other parents in the group sessions? 13. What, if any, benefits, or achievements do you have during the intervention? e.g., Parental skills, parent-child interaction. 14. How could the program have been improved to help you more? 15 What types of families do you think this program might work best for?

#### Qualitative Evaluation: Focus Groups

The purpose of the focus groups was to learn about parents’ perceptions toward this parent coaching intervention based on their 12-week telehealth experience. The focus group allowed researchers to explore the “what” and “why” questions corresponding to the survey questions and foster insights about personal perceptions, technical and social issues and practical experience that parents encountered during the intervention. The focus group followed a semi-structured agenda with 15 prompts based on the same conceptual constructs in the Program Evaluation Survey. Table 3 shows the examples of matching survey questions to focus group prompts. For example, the first question in the focus group was matched with the construct of satisfaction and the fifteenth question was matched with recommendation. Matching was a type of integration procedure in mixed methods research during data collection, where the researcher intended to collect both qualitative and quantitative data about the same domains, constructs or ideas (Fetters, 2019).

Twenty-four of the 32 participants in the intervention study took part in the focus groups. The 24 participants were divided into five groups based on their conditions and availability. In the end, two focus groups were held for seven participants in the self-directed group (comparison), and three focus groups were held for 17 participants in the group therapy group (treatment). Five focus groups were conducted in Chinese online, immediately after the intervention using videoconferencing software, DingTalk. Each focus group lasted about 1 to 1.5 hours, was audio-recorded, and included 2 to 6 parents. The second author (HC) facilitated all five focus groups as a moderator, following the same agenda to ensure that all questions were addressed. The specific prompts are listed in Table 3.

### Data Analysis

Quantitative statistical analyses were conducted using SPSS 28.0. *P*-values below 0.05 were considered statistically significant. Independent *t*-tests and Pearson’s χ^2^ tests were conducted to test the group difference in baseline participants’ demographic characteristics. Program Evaluation Survey data was exported from Qualtrics to run descriptive statistics. All data were reviewed for independence, normality, and homogeneity of variance. Descriptive statistics including frequency, mean, percentages, and standard deviation were computed. Independent samples *t*-tests were conducted to compare parents’ evaluation on the program implementation, perceived competence, and self-efficacy between treatment and comparison groups.

The qualitative data from focus groups were de-identified and transcribed verbatim in Chinese. Thematic analysis was conducted with the overarching research goal of understanding parents’ perspectives based on their 12-week experiences in this telehealth intervention. Those experiences included, but were not limited to, their personal perception of the program, perceived fit of the program in terms of whether it addressed a specific issue or problem, and the extent to which the skills learned can be successfully implemented. We followed the procedures for the thematic analysis approach described by Braun and Clarke (2006), which uses the constant comparative method focusing on abstraction rather than description of data. All the transcripts were read and re-read by the first (LQ) and second authors (HC) to gain familiarity, and initial thoughts were noted separately. The initial codes were generated independently by the first (LQ) and second authors (HC) based on the construct/scope of questions asked in the focus group, discrepancies were addressed through discussion. The preliminary codes were then collated into each potential theme in a systematic manner using a constant comparative method. Six rounds of review sessions were conducted to ensure the data extracts illustrated the analytic themes and identified the subthemes within each theme. Then, two researchers reviewed the themes and subthemes against the original transcripts to reexamine and determine the analysis summarized a convincing and well-organized story about the data and topic. The most relevant quotes were highlighted and extracted from the transcripts, and the quotes were translated in English and reviewed independently by the authors. Lastly, themes, subthemes, and codes were translated into English and reviewed with an English native speaker who is a pediatric physical therapist to ensure accuracy.

## Results

### Participant Characteristics

Participant demographic characteristics were reported in Table 2. No significant group difference was found in other demographic variables at baseline, which indicated that the participants in both conditions have similar demographic characteristics in terms of sex, age, ASD severity. However, the 14 participants in the self-directed condition (*M* = 0.82, *SD* = 0.27) demonstrated a significant lower intervention completion percentage than 18 participants in the web+group therapy condition (*M* = 0.99, *SD* = 0.06) at the post-intervention assessment, *t*(14) = -2.25, *p* = 0.04. The participating families came from 20 provinces, which covers most provincial level administrative region in China (20 out of 32).

### Program Evaluation Survey

Thirty-two parents in the intervention completed the Program Evaluation Survey. Table 4 displayed the descriptive statistics of Skewness and Kurtosis. The scores of *Recommendation* in the self-directed group was non-normally distributed, with skewness of -1.89 and kurtosis of 4.67. The other items in the survey were fairly symmetrical because the absolute values of Skewness and Kurtosis were less than 0.5 or less than 3 times the standard error. The homogeneity of variance was tested through Levene’s test, and no significant results were obtained, assuming no violence in homogeneity of variance.

**TABLE 4:** The skewness and Kurtosis of items in program evaluation survey (*N* = 32).

		Self-directed web (*n* = 14)				Web+group therapy (*n* = 18)			
		Skewness		Kurtosis		Skewness		Kurtosis	
Item		Statistic	Std.Error	Statistic	Std.Error	Statistic	Std.Error	Statistic	Std.Error
1	Satisfaction program content	–0.32	0.60	–2.24	1.15	–1.46	0.54	0.14	1.04
2	Satisfaction telehealth delivery	–0.43	0.60	–0.39	1.15	–0.50	0.54	–1.99	1.04
3	Recommendation	–1.89	0.60	4.67	1.15	–1.26	0.54	–0.34	1.04
4	Acceptability program content	0.03	0.60	0.21	1.15	–0.24	0.54	–2.20	1.04
5	Acceptability telehealth delivery	0.32	0.60	–2.24	1.15	–0.50	0.54	–1.99	1.04
6	Appropriateness program content	0.00	0.60	–2.36	1.15	–0.24	0.54	–2.20	1.04
7	Appropriateness telehealth delivery	0.41	0.60	–0.76	1.15	–0.32	0.54	–1.24	1.04
8	Feasibility program content	–0.32	0.60	–2.24	1.15	–0.24	0.54	–2.20	1.04
9	Feasibility telehealth delivery	–0.11	0.60	–0.86	1.15	–0.62	0.54	–0.39	1.04
10	Program difficulty	0.32	0.60	–0.63	1.15	0.65	0.54	–0.21	1.04
11	Background needed	–0.66	0.60	–1.56	1.15	–0.48	0.54	–0.95	1.04
12	Frequency of learning	0.52	0.60	–0.73	1.15	1.11	0.54	2.60	1.04
13–22	Total perceived competence	–1.16	0.60	0.93	1.15	0.15	0.54	–1.41	1.04
23–32	Total self-efficacy	–1.55	0.60	1.51	1.15	–0.21	0.54	–1.12	1.04
									

**FIGURE 1:**
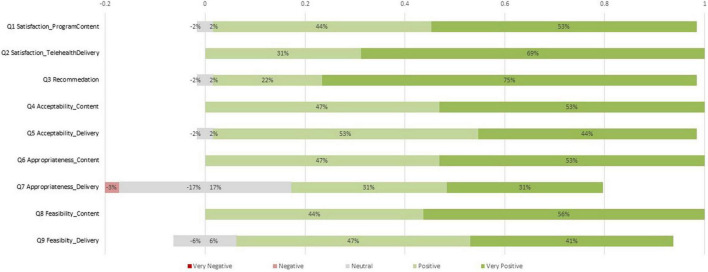
The percentage of ratings on item 1 to item 9 in program evaluation survey.

**FIGURE 2:**
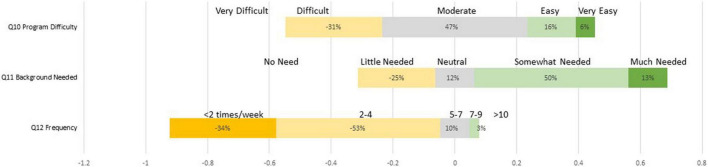
The percentage of ratings on item 10 to item 12 in program evaluation survey.

Table 5 showed the average scores on implementation questions (item 1–12) as well as total scores on perceived competence (item 13–22) and self-efficacy (item 23–32) across groups. The independent-samples *t*-tests were conducted to examine the group differences. There were no group differences on item 1 to 12 regarding parents’ ratings on the program content or telehealth delivery on satisfaction, recommendation, acceptability, appropriateness, and feasibility. According to the findings, both groups held similar perceptions on program implementation. Specifically, Figure 1 showed the percentage of ratings on item 1 to item 9 in program evaluation survey. Except for item 7, almost all parents rated positively for the majority items. There were 11 out of 32 (34%) parents rated neutral and 1 parent (3%) rated negative on Item 7, the Appropriateness of Telehealth Delivery. Figure 2 showed the percentage of ratings on item 10 to item 12. The program’s difficulty level was rated moderate by 47% of participants (15/32) with 16% rating it easy and 6% rating it very easy. 50% of participants (16/32) rated “somewhat needed” on the background knowledge, 25% (8/32) rated “little needed,” and 13% (4/32) rated “much needed.” The majority of participants (17/32, 57%) used the program 2–4 times per week, with 34% (10/32) studying less than two times per week.

**TABLE 5:** Average scores on program evaluation survey items between groups (Mean ± SD).

Item		Self-directed (*N* = 14)	Web+group therapy (*N* = 18)	Total (*N* = 32)
1	Satisfaction program content	4.36 ± 0.63	4.61 ± 0.50	4.50 ± 0.57
2	Satisfaction telehealth delivery	4.57 ± 0.51	4.78 ± 0.42	4.69 ± 0.47
3	Recommendation	4.43 ± 0.68	4.75 ± 0.43	4.61 ± 0.56
4	Acceptability program content	4.43 ± 0.51	4.61 ± 0.50	4.53 ± 0.51
5	Acceptability telehealth delivery	4.21 ± 0.58	4.56 ± 0.51	4.41 ± 0.56
6	Appropriateness program content	4.50 ± 0.52	4.56 ± 0.51	4.53 ± 0.51
7	Appropriateness telehealth delivery	3.57 ± 0.94	4.17 ± 0.79	3.91 ± 0.89
8	Feasibility program content	4.57 ± 0.51	4.56 ± 0.51	4.56 ± 0.50
9	Feasibility telehealth delivery	4.07 ± 0.73	4.44 ± 0.62	4.28 ± 0.68
10	Program difficulty	2.79 ± 0.70	3.11 ± 0.96	2.97 ± 0.86
11	Background needed^∧^	3.29 ± 0.91	3.67 ± 1.08	3.50 ± 1.01
12	Frequency	1.71 ± 0.72	1.89 ± 0.76	1.81 ± 0.74
13–22	Total perceived competence	30.14 ± 14.78	38.72 ± 8.14*	34.97 ± 12.11
23–32	Total self-efficacy	35.00 ± 16.43	41.22 ± 7.30	38.50 ± 12.34

∧reversed score, *p < 0.05.

Parents in the self-directed group (*M* = 30.14, *SD* = 14.78) had significantly lower scores in *Total Perceived Competence* than parents in the web+group therapy group (*M* = 38.72, *SD* = 8.14), *t*(30) = -2.09, *p* < 0.05, and the effect size was median, Cohen’s *d* = -0.75. The negative effect size indicates that the mean in the second group was larger, and the negative effect size indicates that the effect increased the mean when adding treatment. The results showed that there existed a significant difference in parents’ perceived competence between the treatment and the active comparison groups. A median effect size indicated that there may be a moderate treatment effect on perceived competence between groups. However, there was no group difference on the total scores of self-efficacy, *t*(30) = -1.44, *p* = 0.16.

### Focus Groups

Qualitative results including themes, subthemes, and codes (with original Chinese results) were summarized in Supplementary Appendix B. Five overarching themes emerged, most of which reflected constructs in the conceptual framework for implementation outcomes and included suggestions for future research.

#### Theme 1: Acceptability

During the focus groups, parents talked about their personal experience with various dimensions of this web-based program, five subthemes emerged within this major theme: (1) content, (2) design, (3) delivery, (4) participation, and (5) group therapy^1^.

##### Content

Parents across both intervention groups had overall positive perception on the program content, and they particularly commented on the program being professional and systematic with substantial learning materials. 2029 stated, *“The knowledge covered in the program is systematic and the topics are very comprehensive.”* In addition, parents mentioned that the materials covered in the program focused on the key points and was digestible. 2029 added, *“The whole program was presented in a very clear way to me. There are many techniques to use and steps to follow. These were clearly explained and emphasized.”* Furthermore, parents identified their favorite and most challenging techniques for interacting with their children. The most frequently endorsed techniques were presented in Supplementary Appendix B. For example, one parent (1002) stated that the use of gesture worked best in their situation, she stated, *“I found his communication becomes much better after using gestures and his emotional problems get better as well, and I believe that the use of gestures opens a door for him in communication.”* Another parent (2021) mentioned joint attention was her favorite topic, *“It allows me to build a connection between me, my kid, and an object, and this connection is like magic that makes the interaction between me and my child more natural in a harmonious way.”* However, 3135 found joint attention techniques quite challenging, *“If I initiate the joint activities, now he can respond and follow. However, it is challenging for him to initiate and share first. We are still working on that.”*

##### Design

When asked about the program’s design or structure, parents stated that it was well structured and easy to follow, and that the design was innovative, with an acceptable weekly dosage. 1005 stated, *“I really liked the way the instructor breaks down each module into steps, which are very easy to follow. In addition, the duration for learning is about 30 min, which can be finished quickly.”* In addition, parents endorsed the topic sequencing in a learning progression and the structure encouraging participation and self-learning. 1002 stated, *“There are setups in the program which really motivate me to learn, such as the new module unlocks when you finished the prerequisite in previous week.”* The tailored feedback, demonstration and commentary videos, and group discussion were the top three learning components that parents stated as their favorite. For example, 1019 stated, *“The teacher explains why we should do it and how to do it in the commentary videos. She uses lay language and presents the theoretical part in a digestible, easy-to-understand way.”*

##### Delivery

Parents shared their personal perceptions and experience on the program delivery when discussing about the telehealth format of this web-based program. They stated that telehealth delivery increased access to services and professionals, was time-efficient, and provided an alternative method without location or space constraints, particularly during the pandemic. 1002 stated, *“Remote learning is more convenient and timesaving, especially after COVID. It is flexible for parents to arrange time for learning and for practice with the kids.”* The limitations of remote learning were the high demands on the Internet and the reliance on technology. 1018 stated, *“There’s only one computer in the household which is my husband’s. Therefore, I have to study when he is not working.”* Most parents experienced slow downloading speeds when viewing learning materials, and other incidents for smartphone and tablet users included Canvas app crash and video stuck in processing. For example, 2043 mentioned, *“I was really frustrated with the Internet speed; it took forever to download the videos. Then I went to upgrade my broadband and problem solved.”*

##### Participation

Parents felt empowered and strengthened when they realized they could integrate techniques learned each week into their children’s daily activities. It did, however, take some time for parents to implement the techniques with their children, and some reported that the rate of progression differed between themselves and their children. For example, 3233 mentioned, “… *the kid does not always respond like a textbook or respond as you expected. I need to repeat the skills in the previous week so that I can use newly introduced strategies to interact with him.”* Parents encountered numerous situations when submitting homework, the most commonly described ones were children unwilling to participate, target behaviors hard to capture, and failure practice not being reviewed. For instance, 2036 stated, *“There are times that I cannot record the video as I planned. Sometimes it’s because I cannot proficiently use the skills, sometimes it’s the child not being cooperative.”* However, majority of parents stated that homework assignments helped them reflect on the skills and techniques learned in the program, and that tailored feedback was useful and help guide learning. For example, 1002 stated, *“The feedback is very informative, and I benefit a lot from that.” 1019 stated, “I can go back and watched it (homework) with the comments from the teacher …And this is the biggest takeaway from the program.”* The main reason for not submitting homework videos was a lack of family support. For example, 1018 stated, *“It’s very difficult for me to submit the video homework because I am the only parent in the household.”*

##### Group Therapy (see Text Footnote 1)

This subtheme was generated based on prompts solely for parents in the treatment group (17 out of 24). They indicated the group therapy sessions relieved stress and created an interactive learning environment where they received direct coaching from the therapist and interacted with peers. For example, 3131 stated, *“We have a scheduled time spot every week …I believe this is a core strength that online group session holds compared to the one-to-one therapy because it helps every family in the group to relieve their parenting stress at different levels.”* 2036 stated, *“The feedback that I got from group discussion session was splendid.”* Many parents stated that they felt committed to participating in the group session, with guided reflection, peer commenting, and role-playing receiving the most votes as their favorite group activities. For example, 3230 stated, *“I know things that I need to accomplish within the week, which gave me lots of motivation. The group session is great for me and keeps me in tune and updated.”* 2021 mentioned, *“The teacher made a live demonstration, and then each parent played a role and practiced the skills online. I was really overwhelmed because I did not know the virtual group can be interactive and highly engaged.”*

#### Theme 2: Appropriateness

A second theme that emerged was the perceived fit of the web-based program to address a specific problem or to achieve a specific purpose. Five subthemes emerged: (1) family centered care, (2) home-based intervention, (3) strategies relative to daily activities, (4) remote learning platform, and (5) program-based community (see text footnote 1).

##### Family Centered Care

According to the parents, this web-based program provided an opportunity for all family members in the household to participate. For example, 3131 stated, *“My biggest takeaway is the environment that we built as a family and its impact on the child, because we REALLY are seeing his progress every week.”* Many parents commented that the intervention skills and techniques emphasized parent-child interaction and the program emphasized parent mental health. For example, 1014 stated, *“My arms had a lot bruises that his bites as he attacked a lot, I was very anxious back then. But now, we are very close and intimate, our relationship is influenced the most.”* Meanwhile, parents reported that their interaction with their children after work was limited. As 2026 mentioned, *“We have two kids*… *Sometimes the kid could already be asleep by the time I get home. It is challenging.”*

##### Home-Based Intervention

Parents stated that this web-based program was a good fit since materials or skills provided could be used at home, and the self-directed program was flexible without time or location limits. Many parents indicated the remote learning was advantageous since it saved time and money that would have been spent on travel. For example, 3227 stated, *“The remote learning is time-saving. We don’t have to take the kid to the institution …it is really convenient.”* Furthermore, parents stated that they had never considered home as a possible intervention site, and their attitude toward early intervention shifted from a heavy reliance on local resources to a more balanced partnership between family and professionals. For example, 3233 stated, *“I used to believe placing the kid in an institution that works 24 hours a day was the best solution, but I don’t have the financial ability to do so. I now realize I can interact with my kid with skills, and he trusts me more.”* However, several parents experienced interruptions during intervention, such as business trips, being sick or other life responsibilities. For example, 1018 stated, *“I take care of my child all by myself. I had a fever once and had trouble concentrating on any task.”*

##### Strategies Relative to Daily Activities

Parents viewed this web-based program suitable because they learned intervention skills through the life events, and these intervention strategies could be integrated into their child’s daily routine to intervene. For example, 2029 stated, *“All the skills enable me to have a better understanding of my child. I often use steps and skills to plan out our daily activities now.”* Many parents mentioned the child-led mindset was fitting to promote the parent-child relationship. It also aided in better understanding the motivation behind the child’s behaviors. For example, 1019 stated, *“The ESDM is more like a mindset to me. It has a good concept, to follow the child’s lead. You should start with his interests and incorporate that into his daily routines and games.”*

##### Remote Learning Platform

The web-based program used Canvas to distribute learning materials. Parents valued this program because it supplemented the limited local resources and allowed them to access the materials repeatedly as needed. For example, 3135 stated, *“I can arrange time to study the learning materials because we can watch these videos repetitively.”* 3233 stated: *“I think remote learning is extremely awesome because we can get in touch with American experts.”* However, most parents had negative experiences with the Canvas app or video streaming to varying degrees, and many complained that remote learning lacked in-person interaction and could not provide instant feedback. As 3227 mentioned, *“The only thing that I am not satisfied with is the difficulty of using the APP. The DingTalk meetings sometimes get very laggy …but it is common for remote learning.”* 1020 also mentioned that the communication was not timely, she stated, *“I gave some feedback on the software problem at the beginning of the program and I had to wait for the next day to get a response. The problem could not be solved in time due to the time difference.”*

##### Program-Based Community (see Text Footnote 1)

Parents, in the treatment group, viewed the online group therapy as efficacious for creating a program-based community where they provided social-emotional support, shared personal experiences, and reduced stress and anxiety. For example, 2029 stated, *“After discussing with other parents, I find comfort in the fact that everyone is in the same situation, and that means I am not bad at taking care of my child.”* 3233 mentioned, *“I feel less anxious when I get to know other moms with older kids. I feel it is ok that my kid doesn’t have some skills now, I believe he will gradually get it, just like other kids.”*

#### Theme 3: Feasibility

Parents discussed the components that enabled them successfully to implement the intervention skills with their child. Five subthemes emerged: (1) parent modeling, (2) step by step instruction, (3) learning by doing, (4) formative and tailored feedback, and (5) peer learning (see text footnote 1).

##### Parent Modeling

Many parents stated the demonstration and commentary videos facilitated them to carry out the intervention skills at home. Especially through parent modeling, they found it doable and intuitive to observe and learn. For example, 1019 stated, *“I will not know how to practice the strategies out if I only watched the lecture videos. The combination of lectures and demonstration videos makes the intervention a relatively easy and doable task.”* 3227 also mentioned, *“You can observe directly what techniques other parents use in demonstration videos.”*

##### Step by Step Instruction

One of the practical reasons for the success of the intervention strategies was the step by step instructions. It guided parents to interact with their child and perform the intervention strategies. For example, 3135 stated, *“There was a practice manual for each module, it tells us what to do at each step.”*

##### Learning by Doing

Many parents indicated this web-based program emphasized the application of intervention skills and strategies through daily routine activities or play. For example, 1002 mentioned, *“This program focuses more on social interaction strategies which can be used in parent-child interaction at home.”* They expressed that skills learned in the program was easy to apply and implement as most situations occurred in a naturalistic setting. 2029 stated, *“Without the demonstration videos, I would never know there are strategies which can be used when putting on shoes, putting on clothes, even when eating meals together.”*

##### Formative and Tailored Feedback

Tailored feedback was one of the program components that most parents praised. Parents stated that the feedback facilitated them to complete the program and implement the strategies in a relatively easy way. For example, 3226 stated, *“After reading the feedback, I know where I did well and why it worked, also what I lacked, I will try to improve that for next time.”* They also indicated that the tailored feedback provided them with relevant information or cues to guide them toward a possible solution given their existing personal resources and circumstances, rather than just a simple judgment of whether their performance was correct or not. A few mentioned the feedback was delayed due to the nature of the program structure. 3228 stated: *“My practice feels different given the feedback I got. It made me understand my child’s behavior and reflect on my own behaviors.”*

##### Peer Learning (see Text Footnote 1)

Peer learning experience was another subtheme emerged from responses within parents in the treatment group. They valued the group session because it allowed them to share ideas and learn through group discussion and group activities. For example, 3135 stated, *“At the reflection section, the parents comment on each other’s homework, which is a great learning activity. We are able to learn from others’ strengths.”*

#### Theme 4: Project-Level Suggestions

During the focus groups, we asked about how the program could be improved to encourage participation, and parents responded with suggestions based on their own experiences and needs. Three subthemes emerged: (1) personalized pace, (2) advanced program in need, and (3) group-shared platform for maintenance.

##### Personalized Design

Although parents navigated the program at their own pace, many expressed their preferred progression. Some wished that the first few weeks be extended so that they could practice more, while others wished that the content in the latter weeks be given more time. For example, 1002 stated, *“…could we extend the module? For example, we could have divided 1 week into two and studied more thoroughly.”* 3131 suggested, *“The instructor could gradually step back from a central role. In this case, we, parents, not only learn theoretical concepts, but master these techniques in a real-world setting.”* Some parents suggested having case study opportunities to address a specific problem they encountered in their lives, such as bedtime routines, mealtimes, and challenging behaviors. 3233 stated, *“How about the group session solely focused on one family, we could discuss everything about the child, such as his functioning level, what he can and cannot do, and then propose intervention strategies for the mother?”* Parents in the treatment group proposed a grouping strategy based on children’s levels of functioning, so that the session’s topic could be packed together. Some parents also suggested a live stream coaching session in which parents and children interact in front of the camera and receive feedback. 2036 stated, *“Perhaps the courses could be assigned to parents based on their child’s level of functioning.”*

##### Advanced Program in Need

The advanced program was in high demand among the parents, particularly those in the treatment group. Several parents inquired about a motor or language-specific program, while others suggested that more information on adaptive skills in the future. As 2023 stated, *“My child’s receptive and expressive language skills have greatly improved, but there’s still room for improvement in daily living skills. I wish there was more content focusing on that.”* Parents expressed their needs for an in-person program and group therapy for their children. For example, 3131 stated, *“We would also like to see an improved in-person version of this remote-learning program. If possible, I believe our family would benefit greatly from this.”*

##### Group-Shared Platform for Maintenance

In addition to extending access, parents in the treatment group suggested establishing a shared platform for practice videos or their own toy collection. 3124 stated, *“I wish there was a way for us to share with individual accounts so that we could watch each other’s videos.”* Parents suggested that the program groups be kept for follow-up and additional group therapy sessions be held. 3227 stated, *“I wish we could keep talking for another 2 or 3 months after the program is over. stay in touch with the instructor and each other.”*

#### Theme 5: Service-Level Considerations

Parents highlighted their success and accomplishments in the focus groups, and the discussion expanded beyond project implementation, from an individual to a service level. Three subthemes that most connected to service outcomes emerged: (1) cost, (2) timeliness, and (3) perceived effectiveness.

##### Cost

Even though the current study was funded, parents expressed a willingness to pay for telehealth services, particularly high-quality services. 1019 stated, *“I am willing to pay. Nowadays we all know that you must pay for knowledge. It’s worthwhile if the expertise and service are decent.”* Some parents reported that they could detect the difference between high- and low-quality services after the intervention, and the cost for equivalent services relied on the therapist-to-patient ratio and the therapist’s qualification. 3233 stated, *“When it came to the cost, I always evaluate if the treatment session is one-on-one or one on many. Our group has a one to six ratio, which is acceptable.”*

##### Timeliness

Another subtheme emerged on the service level is the timeliness. Most parents would recommend such a program to newly diagnosed families, and to families with young children aged 2–5. 1002 stated, *“If I had the chance, I would’ve enrolled my child in the program when he was younger, and I would strongly recommend it to younger kids. I feel like time is more valuable when we integrate strategies in the play to promote his development.”* Parents noted the remote learning could assist underprivileged families receive timely treatment, and they recognized the potential of adopting ESDM in the neural typical family as well. 2023 stated, *“Many clinicians, including therapists, in the second-tier city here at southwest China are unfamiliar with ESDM. I also met speech therapists who do not heard of ESDM. This online program gave me access.”* 2043 added, *“I believe that most families, including NT families, require such a program because parents are too busy working to educate and raise their children.”*

##### Perceived Effectiveness

Parents shared their perceived changes during the focus groups, and they indicated how much they learned about ASD and how their attitude toward ASD intervention changed. 3228 stated, *“I did not know anything about ASD. I started to learn about Autism while watching other kids’ behaviors in the demonstration videos.”* 1002 stated, *“I feel that once I began to use the program’s strategies or skills, parenting became easier for me.”* Parents also highlighted individual changes, such as changes in child behavior, parenting skills and parent-child interaction. For example, 1018 stated, *“I learned how to interact or play with my child in a natural living environment, how to follow him and set up the joint activity*… *I feel he progressed tremendously.”* 2021 stated, *“I love my kid more because I discovered something subtle in her behaviors which I missed before. I understand her better now.”* 2023 stated, *“My child improved greatly over the past 3 months. He could only say ‘hua’ and ‘shi’ on September 7th, but now he can speak sentence, count numbers, even recite poetry. Great improvement.”* Parents also mentioned parental self-efficacy and mental health improvement. 3131 stated, *“Now I can see his progress from the beginning, and I can identify how he improves and appreciate his progress. It is the parenting mindset that changed most.”*

### Mixed Methods Findings

The mixed methods findings occurred by merging the quantitative and qualitative results. A more complete understanding of the research question was achieved by relating the conceptualized themes and subthemes to interpret and develop inferences between the quantitative and qualitative findings. The joint display provided descriptive statistics of the program evaluation with the qualitative themes and subthemes from the focus group, and the meta-inferences are reported accordingly in Table 6.

**TABLE 6:** Joint display of the quantitative and qualitative findings with meta-inferences.

Quantitative (Mean ± SD)		Qualitative		Meta-inferences
		
		Themes	Subthemes	Interpretation of mixed methods findings
4.53 ± 0.51	Program content	Acceptability	Content Design Delivery	The subthemes suggested various aspects of the program was acceptable for parents based on their personal experience and perception. It was confirmed by the high average scores in both items under *Acceptability*.
4.41 ± 0.56	Telehealth delivery		Participation	
			Group therapy^†^	

4.53 ± 0.51	Program content	Appropriateness	Family centered Care Home-based Intervention Strategies Relative to Daily Activities	The subthemes represented how this program met parents’ needs or addressed their problems. Comments in *Remote learning platform* was relatively neutral due to technical issues which confirmed a lower average score in the telehealth delivery item, compared to program content item, under *Appropriateness.*
3.91 ± 0.89	Telehealth delivery		Remote learning platform	
			Program-based community^†^	

4.56 ± 0.50	Program content	Feasibility	Parent modeling Step by step instruction Learning by doing	The subthemes listed all practical elements that made this program implementable for parents. It was confirmed by the high average scores in both items under *Feasibility*.
4.28 ± 0.68	Telehealth delivery		Formative and tailored feedback	
			Peer learning^†^	

N/A	N/A	Project-level suggestions	Personalized design Advanced program in need Group-shared platform for maintenance	This qualitative theme complemented the survey outcomes with expansions on suggestions for future program.

N/A	N/A	Service-level considerations	Cost	The subtheme complemented the survey outcome on cost for similar service.
4.61 ± 0.56	Recommendation		Timeliness	This subtheme confirmed with the average score under *Recommendation* with detailed family profiles. This subtheme complemented the survey outcomes with expansions on the effectiveness of intervention. The subtheme partially related to the outcomes on parents’ perceived competence and self-efficacy with a broader non-overlapping aspects, such as attitude toward such service.
34.97 ± 12.11	Perceived competence		Perceived effectiveness	
38.50 ± 12.34	Self-efficacy			

*^†^Subthemes emerged from responses within parents in the treatment group (web+group therapy).*

Overall, the two types of findings related to each other with regards to the majority of implementation outcomes, such as acceptability, appropriateness, and feasibility. The mixed methods findings showed expansion with central overlapping commonality as well as broader non-overlapping interpretations. Specifically, *Project-level suggestions* complemented the survey by offering detailed suggestions for future program. In addition, *Service-level considerations* partially related to the survey on the *recommendation* and parents’ *perceived competence* and *self-efficacy*, but complemented the survey on *Cost* and *Perceived Effectiveness of Intervention*.

## Discussion

The current study aimed to investigate Chinese parents’ experience of participation in a 12-week group-based parent coaching intervention based on the P-ESDM and delivered *via* telehealth. We employed a mixed-methods approach using the post-intervention survey and focus groups to evaluate the program implementation from parents’ perspectives. The mixed methods findings provided detailed understandings of the elements or ingredients that determined the implementation success in this culturally adapted program. It revealed (1) the elements of this program that were acceptable to parents, such as program content, design, delivery, participation, and group therapy; (2) how this telehealth program met parents’ needs or addressed their problems (*via* family centered care, home-based intervention, strategies relative to daily activities, remote learning platform, and program-based community); and (3) what practical elements made this program feasible for parents to implement, such as parent modeling, step-by-step instruction, learning by doing, formative and tailored feedback, and peer learning. Findings of this study have implications for better understanding of the telehealth delivery of evidence-based autism-related services among Chinese parents, such as P-ESDM. Results are discussed below.

Overall, parents across groups rated the elements of program implementation (i.e., satisfaction, recommendation, acceptability, appropriateness, and feasibility) positively. Most parents rated the program’s difficulty levels as moderate, believing that there was some background knowledge required for participation. The majority of parents used the program less than four times per week. Despite the fact that parents in the treatment group had significantly higher scores in total perceived competence on the skills gained during the intervention, there was no group difference in self-efficacy. The observed increase in total perceived competence in the treatment group could result from the group therapy itself. Another possible explanation for this might be that fewer people in the self-directed group completed the intervention compared to the treatment group, with the intervention completion ratio 57 to 94%, respectively. However, the findings suggest that parents in both groups had a comparable level of confidence in their ability to carry out the intervention skills after the program, even though the self-directed group had lower total perceived competence scores and fewer people completed the intervention. Self-efficacy was considered as a predictor of health behavior change and maintenance (Rosenstock, 1974; Champion and Skinner, 2008). According to Strecher et al. (1986), health care professionals can influence self-efficacy, and changes in self-efficacy were associated with changes in behavior. Taken together, these findings indicate that parents, whether they participated in group therapy or not, had a similar chance of initiating and maintaining health behavioral change after this telehealth program.

Telehealth created opportunities to deliver acceptable and appropriate services for children with disabilities, particularly during the COVID-19 pandemic (Camden and Silva, 2021; Rao, 2021). Our findings indicated that Chinese parents showed a high level of satisfaction and acceptability to this modified group-based parent coaching program based on P-ESDM. Moreover, the *program content, design, delivery, participation, and group therapy* were five aspects that parents considered when evaluating the program’s acceptability. The evaluation was mainly based on whether their needs, preferences, or expectations differed (Proctor et al., 2011). Specifically, *tailored feedback, demonstration and commentary videos, peer commenting, live coaching, and guided reflection* were top five telehealth strategies strongly endorsed by the majority of Chinese parents. With opportunities in pediatric telehealth created by the COVID-19 pandemic for families of children with disability (Camden and Silva, 2021), therapists need to learn more about acceptable telehealth strategies and adopt them in order to engage families in exploring how telehealth can best meet their needs.

Despite the numerous advantages of telehealth services, there are still challenges to overcome. Firstly, the availability of equipment and a stable Internet connection are key factors for the implementation of telehealth interventions. Although telehealth interventions have been successfully implemented in rural areas (Parsons et al., 2017; Guðmundsdóttir et al., 2018), barriers to adopting telehealth services continue to exist globally (Scott Kruse et al., 2018). Secondly, difficulties related to technology and infrastructure might restrict the benefits of telehealth services. Some studies have documented technological challenges, such as audio delays, dropouts, and audio or video lag during videoconferencing (Banbury et al., 2018). In our study, the shortage of digital devices, App compatibility, slow Internet speeds, and stream latency were negative technology issues experienced by parents during the intervention. This could be a possible explanation for a relatively lower average score (3.91 ± 0.89) in item 7, the *Appropriateness of Telehealth Delivery*, compared to other items in the program evaluation survey. Thirdly, future research or service providers need to explore strategies to promote parent engagement and completion for telehealth interventions. McGarry et al. (2020) reported a low completion rate, with only four of the eleven enrolled parents completing all five online learning modules. Ingersoll and Berger (2015) found that both self-directed and therapist-assisted groups had high levels of parent engagement and satisfaction with ImPACT program, but having a therapist increased engagement and resulted in a higher completion rate. The same pattern was found in our study, a higher proportion of parents in the web+group therapy group (94%) completed the online program compared to self-directed group (57%). This might suggest that a low dosage assistance from therapists may support parents’ engagement and completion. Another ethical concern in the usage of telehealth is patient privacy and information security, which requires therapists following regional telehealth guidelines and policy and paying attention to ethical issues to provide a safe telepractice.

The program’s appropriateness and feasibility were critical when delivering early intervention *via* digital service because it required extensive parental involvement. As Rao (2021) described the paradigm in pediatric service delivery shifted from a home-based model to a family centered approach, parents became the person who implemented the intervention (Oono et al., 2013). Parents judged appropriateness from a technical or social standpoint based on whether their problems were solved, or their goals were met. Our finding indicated that P-ESDM was suitable for Chinese families with a child newly diagnosed with ASD. Parents were able to integrate *home-based strategies* into *daily activities*, and the *remote learning platform* increased the accessibility to both services and professionals. Long work hours, unexpected interruptions from life events, a lack of in-person observation, and a lack of face-to-face communication were barriers in this program. Feasibility was evaluated by practical experience of parents if a task can be performed relatively easily (Proctor et al., 2009, 2011). The combination of the asynchronized lectures, demonstration and commentary videos in a video-based platform enabled parents to learn the skills through *modeling* and *instructions*. The *formative and tailored feedback* corrected and guided parents’ practice. In addition, the synchronized videoconferencing group sessions provided parents with peer learning experience. All the components mentioned above helped parents successfully implement intervention techniques with their child at home.

Group-based therapy provided psychosocial support for parents of a child with ASD (Farmer and Reupert, 2013), and videoconferencing was an acceptable and feasible approach of delivery for group-based interventions in this population (Parsons et al., 2017; Lodder et al., 2020). In a recent systematic review (Sutherland et al., 2018), parent satisfaction was reported as an outcome variable in nine of fourteen studies, and only three studies were intervention design *via* telehealth, with all three reporting a high level of satisfaction (Ingersoll and Berger, 2015; Hepburn et al., 2016; Pickard et al., 2016). However, none of these three studies were conducted in a group setting. An in-person parent program was conducted in a group basis, and parents reported that the main benefit of the program was “meeting other parents having similar experience and sharing ideas” (Williams et al., 2020). Peer bonding and support were critical for parents of a child with ASD, particularly those who were newly diagnosed. Parents in Lodder et al. (2020) reported that the advantages of videoconferencing outweigh the disadvantages and suggested that a hybrid format is best. More importantly, the home-based telehealth was reported as the lowest cost when compared to in-home therapy and clinic-based telehealth format for ABA service (Lindgren et al., 2016). Overall, this study was one of the few studies which provide parent coaching intervention in a group-based setting *via* videoconference to parents of children with ASD. Our findings demonstrated that the telehealth delivery format was acceptable to Chinese parents. Despite certain suggestions and concerns for better serving parents’ needs, this group-based parent coaching intervention might be a successful program and a promising method of delivery for a low-resource population, such as Chinese parents.

Group-based programs delivered by telehealth would potentially become a more affordable option for Chinese families with a child diagnosed with ASD. According to Guo (2014), only 13.8% of parents considered the cost of their child’s rehabilitation or education as affordable, 55% of parents were satisfied with the professionals or the service provided. Moreover, over half of 3,867 families studied (54%) indicated that one parents had to give up work due to their child’s ASD diagnosis. This condition undoubtedly increased financial risk for families who were already in crisis. In our study, 78% of families (25/32) had a monthly family income of less than $3,000. Perhaps not surprisingly, *cost* emerged as a subtheme that complemented the survey outcomes. For example, parents stated the remote program was cost-efficient for them. Limited availability of services in the healthcare and educational systems and a pervasive shortage of resources are key factors in the financial burden faced by parents (Sun et al., 2013a). Parents reported in our study that they faced additional expenses as they explored available healthcare resources, for example, costs associated with traveling to another city for services. According to a recent qualitative study, the fidelity of online parent training during the COVID-19 pandemic in China was limited by inexperienced teachers, and tensions between inexperienced teachers and parents demanded more culturally relevant instructional materials (McDevitt, 2021). Under such circumstances, a well-structured, culturally adapted parent program *via* telehealth may offer a promising solution. More research is needed to determine the feasibility and effectiveness of such programs or instructional materials across a variety of contexts.

The mixed methods findings provided suggestions for improving future iterations of the program. *Project-level suggestions* and *service-level considerations* complemented the quantitative results by emphasizing the need to personalize treatment to optimize outcomes and proposing a potential solution to establish a group-shared platform for longer sustainability. Those results indirectly reflect there is a great need for high-quality early intervention services in mainland China. Future studies should include components or strategies to improve parent engagement and participation, such as practice manuals, homework, and tailored feedback. Such programs should be adjustable in the difficulty level with an acceptable and a manageable dosage to avoid putting additional strains on parents. Furthermore, strategies and methods that can be used to advance personalized interventions need to be further investigated to better serve children at different stages of development and families with diverse characteristics.

## Limitations

There were several limitations in the current study. Firstly, despite efforts to recruit families from a variety of demographic profiles, most participating families included parents with a college degree or higher. The telehealth program excluded those families who did not have access to the internet or digital devices. Second, the sample size is relatively small. However, given this culturally adapted telehealth intervention was newly developed and still on feasibility testing, the current mixed-methods findings provided enriched and comprehensible information about the program implementation compared to solely quantitative ratings. Although it is typical for a convergent design to have the qualitative sample nested within the quantitative sample, most responses from the focus groups came from parents who completed the intervention. We were able to invite one parent from each group who did not complete the program to contribute their views. There was a lack of information from those who did not complete the program or dropped out. More research is needed to discover why some parents dropped out or did not finish on time. Future telehealth research should consider including web-activity data to gain a better understanding of digital service usage and intervention dosage, such as the number of unique views and minutes viewed on intervention videos. Furthermore, for future research, a user-friendly learning platform and Internet access may be critical factors in telehealth program participation.

## Conclusion

The current study used a mixed methods approach to better understand parents’ perceptions of the implementation outcomes of this modified Chinese version of P-ESDM delivered *via* telehealth. According to our findings, parents in both self-directed and web+group therapy groups had positive perceptions in program satisfaction, acceptability, appropriateness, feasibility, and recommendation. Both groups reported high parent perceived competence and self-efficacy, and group-based parent coaching session increased parent perceived competence. Findings indicate the application of telehealth was acceptable, appropriate, and feasible for Chinese parents. It may suggest the possibility of using telehealth as a service delivery option. In addition, group-based parent coaching intervention could be a promising home-based service model to increase parental perceived competence. Future research should continue to identify culturally appropriate PMI for parents in low-resource contexts, and a large scale RCT is needed to investigate the effectiveness of group-based PMI *via* telehealth. More studies are urgently needed to develop and evaluate the feasibility and effectiveness of this type of intervention during and after the COVID-19 pandemic in resource-limited communities.

## Data Availability Statement

The original contributions presented in this study are included in the article/Supplementary Material, further inquiries can be directed to the corresponding author.

## Ethics Statement

The studies involving human participants were reviewed and approved by University of Michigan Health Sciences and Behavioral Sciences Institutional Review Board. The patients/participants provided their written informed consent to participate in this study.

## Author Contributions

LQ conceptualized and designed the study, analyzed and interpreted the data, and drafted the manuscript. HC collected the qualitative data, analyzed, and interpreted the results. AM and CC conceptualized and designed the study. CC, WC, HM, and DU oversaw the fieldwork and data collection and interpretation. All authors revised the manuscript and approved the final version of the publication.

## Conflict of Interest

The authors declare that the research was conducted in the absence of any commercial or financial relationships that could be construed as a potential conflict of interest.

## Publisher’s Note

All claims expressed in this article are solely those of the authors and do not necessarily represent those of their affiliated organizations, or those of the publisher, the editors and the reviewers. Any product that may be evaluated in this article, or claim that may be made by its manufacturer, is not guaranteed or endorsed by the publisher.

## Footnotes

1 Subthemes emerged from responses within parents in the treatment group (web+group therapy).
