# Supplementary material for: Assessing the Satisfaction and Acceptability of an Online Parent Coaching Intervention: A Mixed-Methods Approach
Source: Front Psychol. 2022 Jul 28;13:859145. doi: 10.3389/fpsyg.2022.859145 (PMC9367480; doi:10.3389/fpsyg.2022.859145)
Supplement: Supplementary file 1 [file Data_Sheet_1.PDF]

Supplementary Material

Appendix A: Program Evaluation Survey Questions

1. What is your overall satisfaction with program contents of this web-based program?
   - Very Dissatisfied
   - Dissatisfied
   - Neutral
   - Satisfied
   - Very Satisfied

2. What is your overall satisfaction on the Telehealth delivery of this web-based program?
   - Very Dissatisfied
   - Dissatisfied
   - Neutral
   - Satisfied
   - Very Satisfied

3. Would you recommend this web-based program to families with the same needs?
   - Very Unlikely
   - Unlikely
   - Neutral
   - Likely
   - Very Likely

4. I like the content of this web-based program.
   - Very Disagree
   - Disagree
   - Neutral
   - Agree
   - Very Agree

5. I feel that the Telehealth delivery of this web-based program is appealing to me.
   - Very Disagree
   - Disagree
   - Neutral
   - Agree
   - Very Agree

6. I think that the content of this web-based program seems applicable to my situation.
   - Very Disagree
   - Disagree
   - Neutral
   - Agree
   - Very Agree

7. I think the Telehealth delivery of this web-based program seems like a good match to me compared to the in-person format.
    - Very Disagree
    - Disagree
    - Neutral
    - Agree
    - Very Agree

8. I think the skills learnt from this web-based program seems implementable to daily life.
    - Very Disagree
    - Disagree
    - Neutral
    - Agree
    - Very Agree

9. I think it is feasible to participate this web-based program through Telehealth delivery.
    - Very Disagree
    - Disagree
    - Neutral
    - Agree
    - Very Agree

10. Please rate the difficulty level of this web-based program?
    - Very difficult
    - Difficult
    - Neutral
    - Easy
    - Very easy

11. How much background information or knowledge do you think is needed to learn this web-based program?
    - Very little needed
    - Little needed
    - Neutral
    - Somewhat Needed
    - Much needed

12. How often do you study this web-based program every week?
    - <2 times/week
    - 2 - 4 times/week
    - 5 - 7 times/week
    - 7 - 9 times/week
    - >10 times/week

13. How much do you think you have improved by learning the relevant intervention skills each week?

Topics	Not improved at all 1	2	3	4	Greatly improved 5
WK2- Increasing the child's attention					
WK3- Sensory social routines					
WK4- Dyadic engagement					
WK5- Nonverbal communication					
WK6- Imitation					
WK7- ABC's of learning					
WK8- Joint attention					
WK9- Play					
WK10- Pretend play					
WK11- Speech development					

14. How confident are you to use the following intervention skills after the program?

Topics	Not confident at all 1	2	3	4	Very confident 5
WK2- Increasing the child's attention					
WK3- Sensory social routines					
WK4- Dyadic engagement					
WK5- Nonverbal communication					
WK6- Imitation					
WK7- ABC's of learning					
WK8- Joint attention					
WK9- Play					
WK10- Pretend play					
WK11- Speech development					

Questions in Chinese:

1. 请问您对本研究课程相关教学内容整体满意程度?
2. 请问您对本研究课程远程形式（线上授课）的整体评价?
3. 请问您是否会将本课程推荐给相同需要的家庭?
4. 我喜欢本研究课程的教学内容。
5. 我认可本研究课程线上授课这种远程教学形式。
6. 我认为本研究课程的教学内容非常实用。
7. 我认为本研究课程的线上授课形式比线下教学形式更适用于我。
8. 我认为本研究课程的教学技巧可运用到日常生活中。
9. 我认为本研究课程采取的线上教学形式可行。
10. 请您对本课程的难易程度打分?
11. 您认为学习本课程是否需要具备相关背景知识?
12. 您每周学习本课程的频率如何?
13. 您认为每周课程所教授的相关干预技巧，通过学习您提高了多少?
14. 课程结束后，您有多少信心继续使用以下相关技巧?
