# Supplementary material for: Assessing the Satisfaction and Acceptability of an Online Parent Coaching Intervention: A Mixed-Methods Approach
Source: Front Psychol. 2022 Jul 28;13:859145. doi: 10.3389/fpsyg.2022.859145 (PMC9367480; doi:10.3389/fpsyg.2022.859145)
Supplement: Supplementary file 2 [file Data_Sheet_2.PDF]

Supplementary Material

Appendix B: ***Qualitative Results of Focus Group Interviews Presented by Themes, Subthemes, and Codes (Chinese and English)***

Themes	Subthemes	Codes	
Acceptability 接受程度	Content 教学内容	1.1.1 Positive Overall Perception	整体感受好
		1.1.2 Professional	教学内容专业
		1.1.3 Systematic	教学内容成体系
		1.1.4 Substantial	教学内容丰富
		1.1.5 Digestible	教学内容可接受
		1.1.6 Focused on Key Points	教学内容重点突出
		1.1.7 Favorite Techniques: Sensory Social Routines (Physical Play), Gestures (Nonverbal Communication), Joint Attention	最喜欢的技能：感觉社交常规活动（身体游戏）、手势（非口语交流），共同注意力
		1.1.8 Challenging Techniques: Joint Attention, Set A Theme for Activities, Follow the Child's Lead	有挑战的技能：共同注意力，制定活动主题，跟随孩子
	Design 教学设计	1.2.1 Clear Structure	教学设计结构清晰
		1.2.2 Easy to Follow	教学设计层次性
		1.2.3 Innovative	教学设计创新性
		1.2.4 Acceptable Dosage	课时合理
		1.2.5 Topics Sequencing in Progression (Easy – Hard)	教学过程难度循序渐进
		1.2.5 Motivating	教学过程激励性
		1.2.6 Favorite Components: Tailored Feedback / Demo & Commentary Videos /Peer Commenting	最喜欢的教学环节：量身定制的反馈、实操及解说视频、小组成员互评
	Delivery 教学实施	1.3.1 Increased Access to Service and Professionals	增加获得专家指导和服务的机会
		1.3.2 Alternative Due to Covid Restrictions	疫情防控下的优质选择
		1.3.3 No Locations & Space Restrictions	教学实施不受地理环境限制
		1.3.4 Time Efficient	教学实施的时间成本低
		1.3.5 High Demands on Internet	对互联网通讯质量要求高
		1.3.6 Technology Dependent	对教学软件硬件要求高
	Participation 学习参与	1.4.1 Empowering and Strength-Based	基于优势激发的参与能动性及赋能
		1.4.2 Effective, Direct Learning-Focused Feedback	基于学习情况的有效反馈
		1.4.3 Progression Rate Different Between Parents and Children	家长和孩子学习进阶掌握情况参差
		1.4.4 Need Time to Practice the Techniques	需要时间练习技巧
		1.4.5 Homework: Reflective Assignment	家庭作业：反思性作业

		1.4.6 Homework: Children Not Willing to Participate	家庭作业：孩子参与意愿低
		1.4.7 Homework: Target Behaviors Not Recorded	家庭作业：没有拍摄到目标行为
		1.4.8 Homework: Failure Practice Not Reviewed	家庭作业：失败的实操无法得到评价
		1.4.9 Homework: Lack of Family Support	家庭作业：缺乏家庭支持
	Group Therapy*<br>小组学习	1.5.1 Alleviate Stress	缓解压力
		1.5.2 Interactive Learning Climate	互动的学习氛围
		1.5.3 Direct Coaching	直接的辅导
		1.5.4 High Engagement	参与度高
		1.5.5 Commitment	忠实度
		1.5.6 Favorite Group Activities: Guided Reflection, Peer Commenting, Live Role Play/Coaching	最喜欢的小组活动：有引导的反思、同学间互评、线上角色扮演、现场直播辅导
Appropriateness<br>适合程度	Family-centered Care<br>以家庭为核心的医疗	2.1.1 Family Involvement	家庭参与
		2.1.2 Parent-Child Interaction and Relationship Building	亲子互动及关系构建
		2.1.3 Focus on Parent Mental Health	关注家长心理健康
		2.1.4 Limited Parent-Child Interaction at Home Due to Work	工作原因导致亲子互动有限
	Home-Based Intervention<br>基于家庭环境的干预	2.2.1 Home Setting	基于家庭情境
		2.2.2 Flexibility	灵活
		2.2.3 Convenience	便捷
		2.2.4 Attitude Changes Towards Intervention	对行为干预的态度转变
		2.2.5 Interrupted by Life Chores	家庭琐事干扰
	Strategies Relative to Daily Activities<br>日常活动相关的干预策略	2.3.1 Teaching and Learning Through Daily Routines	通过日常生活进行教与学的活动
		2.3.2 Child-Led Mindset	孩子主导的干预思维
		2.3.3 Integration in Daily Routine	干预技巧可融入日常生活
	Remote Learning Platform<br>远程学习平台	2.4.1 Learning Material on Demand Available	学习资料的可获得
		2.4.2 Supplement the Limited Local Support	对当地有限资源的补充
		2.4.3 Lack of In-Person Observation	缺乏现场观摩
		2.4.4 Lack of Instant Feedback	缺乏即时反馈
		2.4.5 Accessibility	无障碍
	Program-Based Community*<br>基于项目的共同体	2.5.1 Social Emotional Support	社会情感支持
		2.5.2 Mental Health Promotion	心理健康促进
		2.5.3 Experience Sharing	经验分享
Feasibility<br>可行程度	Parent Modeling<br>家长操作展示	3.1.1 Intuitive	直观
		3.1.2 Doable	可操作
	Step by Step Instruction<br>循序渐进的引导	3.2.1 Strong Parental Involvement	家长参与程度高
		3.2.2 Focused on Parent-Mediated Strategies	关注以家长为中介的干预策略

	Learning by Doing 实践中学习	3.3.1 Naturalistic Teaching	自然场景教学
		3.3.2 Easy to Use	易上手
		3.3.3 Implementable	可执行
		3.3.4 Practice-Based	勤练习
	Formative and Tailored Feedback 规范的定制化反馈	3.4.1 Verification	检验准确
		3.4.2 Elaboration	阐述详尽
		3.4.3 Delayed Feedback	反馈延迟
	Peer Learning* 组内学习	3.5.1 Group Activities	小组活动
		3.5.2 Peers Commenting and Mentoring	同学互评及指导
Project-Level Suggestions 项目层面建议	Personalized Design 个体化设计	4.1.1 Individualized Progression	个性化学习进度
		4.1.2 Problem-Based Learning	问题导向的学习
		4.1.3 Function Centeredness	以功能中心
		4.1.4 Live Coaching	现场直播指导
	Advanced Program in Need 高阶项目需求	4.2.1 Specialized Program (Motor-Specific, Language Promotion, Adaptive Skills Program)	特定项目需求（动作能力项目、语言能力提升项目、适应性技能项目）
		4.2.2 Group Program for Children	面向孩子的小组项目
		4.2.3 In Person Program	线下项目
	Group-Shared Platform for Maintenance 建立分享式平台以维持小组	4.3.1 Extend the Access to The Program	增加参与时长
		4.3.2 Keep the Group	维持学习小组
		4.3.3 More Group Session	增加小组讨论的环节
		4.3.4 Establish Shared Practice Video Library or Toy Collection	建立分享式实操视频库或玩具库
Service-Level Considerations 服务层面考虑	Cost 代价	5.1.1 Willingness to Pay	付费学习的意愿
		5.1.2 High Demand on High Quality Service	对高质量服务的高需求
		5.1.3 Specialist Dependent	对专家教学引导的依赖
		5.1.4 Therapist to Patient Ratio	治疗师与患者比例
		5.1.5 Cost Efficient for The Remote Learning	远程学习的成本效率
	Timeliness 时效性	5.2.1 Newly Diagnosed Family	新进确诊家庭
		5.2.2 Family with Young Children Aged 2-5	2-5岁孩子的家庭
		5.2.3 Family with Low Resource	低资源的家庭
		5.2.4 Family of Child Not on Spectrum	非谱系孩子的家庭
	Perceived Effectiveness 有效性	5.3.1 Knowledge of ASD and Beliefs about Early Intervention	对自闭症早期干预的信念及相关知识
		5.3.2 Individual Stage of Change: Gains on Child, Parent, Parent-Child Interaction	个体变化程度：孩子和家长的进步、亲子关系改善
		5.3.3 Improvement in Self-Efficacy and Mental Health	自我效能和心理健康水平的改善

*: Subtheme was emerged from response from parents in treatment group
